# Development of Immunostaining Protocols for 3D Visualization of Pericytes in Human Retinal Flatmounts

**DOI:** 10.1369/00221554251323655

**Published:** 2025-03-17

**Authors:** Noëlle Bakker, Aïcha A. Croes, Eva Prevaes, Cornelis J. F. van Noorden, Reinier O. Schlingemann, Ingeborg Klaassen

**Affiliations:** Ocular Angiogenesis Group, Department of Ophthalmology, Amsterdam UMC location University of Amsterdam, Amsterdam, The Netherlands; Amsterdam Cardiovascular Sciences, Microcirculation, Amsterdam, The Netherlands; Amsterdam Neuroscience, Cellular & Molecular Mechanisms, Amsterdam, The Netherlands; Ocular Angiogenesis Group, Department of Ophthalmology, Amsterdam UMC location University of Amsterdam, Amsterdam, The Netherlands; Ocular Angiogenesis Group, Department of Ophthalmology, Amsterdam UMC location University of Amsterdam, Amsterdam, The Netherlands; Ocular Angiogenesis Group, Department of Ophthalmology, Amsterdam UMC location University of Amsterdam, Amsterdam, The Netherlands; Ocular Angiogenesis Group, Department of Ophthalmology, Amsterdam UMC location University of Amsterdam, Amsterdam, The Netherlands; Amsterdam Cardiovascular Sciences, Microcirculation, Amsterdam, The Netherlands; Amsterdam Neuroscience, Cellular & Molecular Mechanisms, Amsterdam, The Netherlands; Department of Ophthalmology, Jules-Gonin Eye Hospital, Fondation Asile des Aveugles, University of Lausanne, Lausanne, Switzerland (ROS); Ocular Angiogenesis Group, Department of Ophthalmology, Amsterdam UMC location University of Amsterdam, Amsterdam, The Netherlands; Amsterdam Cardiovascular Sciences, Microcirculation, Amsterdam, The Netherlands; Amsterdam Neuroscience, Cellular & Molecular Mechanisms, Amsterdam, The Netherlands

**Keywords:** flatmount, immunofluorescence staining, pericyte, retina, 3D imaging

## Abstract

Vascular pericytes are widely present across the human body and crucial in regulating vascular flow, permeability, and homeostasis. In the human retina, pericytes are important for forming and maintaining the blood–retinal barrier, as well as for autoregulation of blood flow. Pericyte loss has been implicated in various pathological conditions. Visualization of pericytes by immunofluorescence (IF) staining provides valuable information on pericyte number, morphology, location, and on expression of anatomic and functional markers. However, species-specific differences in pericyte marker expression exist. In this study, we aimed to develop a novel IF co-staining protocol to detect the pericyte markers NG2, PDGFRβ, αSMA, CD13, and RFC1 in human retinal flatmounts. Unlike retinal sections, retinal flatmounts enable 3D visualization of pericyte distribution across the entire vascular network. Key optimizations included tailoring the fixation method, blocking buffer composition and antibody solvent, as well as using jasplakinolide to enhance αSMA detection. Our protocol successfully enabled double staining of NG2 and PDGFRβ, as well as αSMA and PDGFRβ, whereas CD13 and RFC1 expression was not detectable in human retinal flatmounts. This novel 3D IF protocol enhances in situ visualization of human retinal pericytes, enabling accurate studies of their role in vascular health and disease to aid targeted therapy development.

## Introduction

Pericytes play a crucial role in the formation and maintenance of the inner blood–retinal barrier (iBRB) and blood–brain barrier (BBB). Pericyte dysfunction or loss is a hallmark of a wide range of diseases including Alzheimer’s disease, diabetes-induced microvascular diseases throughout the body, such as diabetic retinopathy (DR), macular edema, and various infectious diseases.^1–4^ Changes in pericyte functions during pathological conditions are reflected in altered marker profiles.^5^ Therefore, we aimed to identify reliable pericyte markers. Pericyte markers identified in this study will ultimately be used in immunofluorescence (IF) staining to study pericytes under pathological conditions. Important to highlight is that most previous studies have been performed in animal models and knowledge about pericyte marker expression in the human retina is limited.

**Figure 1.**
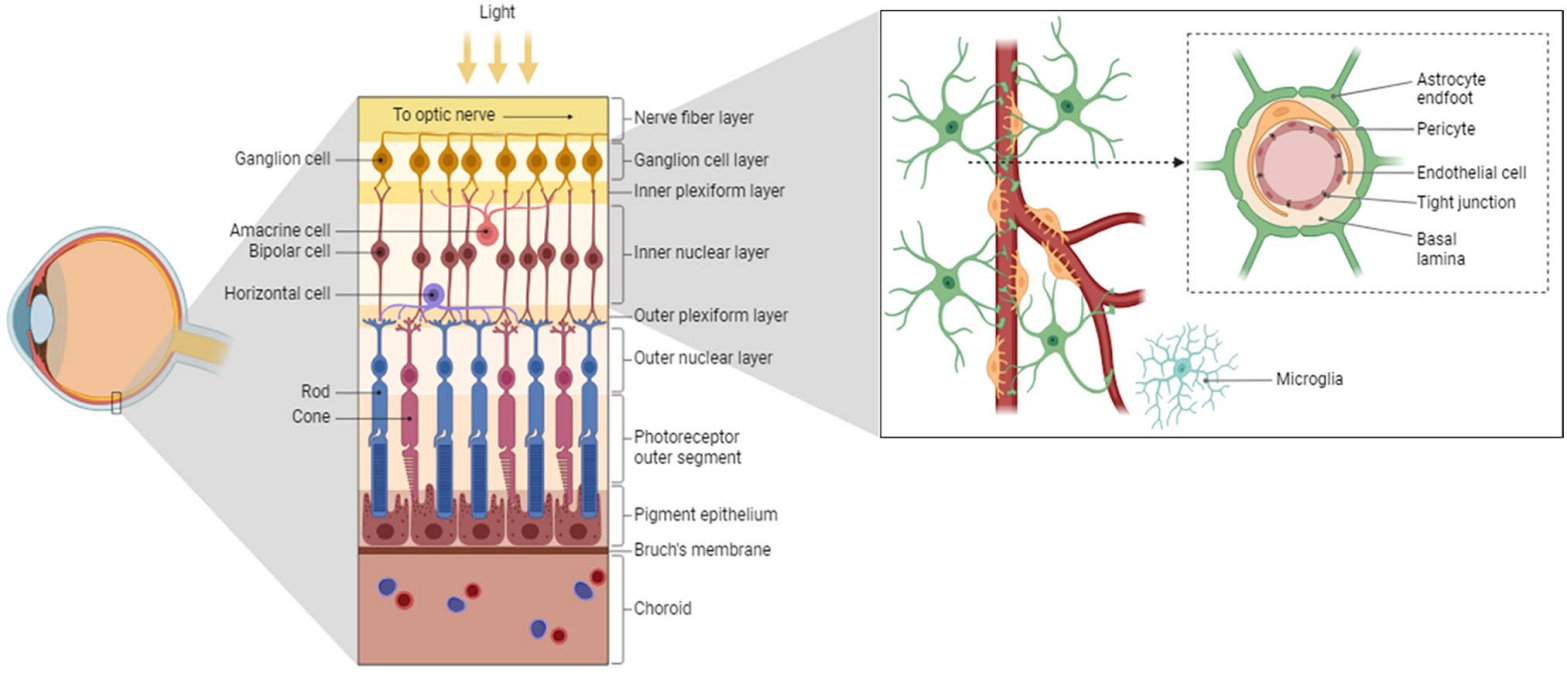
Retinal morphology and structure of the neurovascular unit of the inner blood–retinal barrier. The left panel shows the retinal layers. The microvasculature in the inner retina forms the inner blood–retinal barrier (iBRB; right panel). The iBRB protects the neuronal layers in the retina and consists of endothelial cells forming capillaries in the retina as part of the neurovascular unit. This unit also includes pericytes sharing the basal lamina with endothelial cells, microglia, astrocytes projecting their end feet onto the capillaries, and surrounding neurons. Created with BioRender.com.

Pericytes are mural cells embedded in the microvascular wall and are important in vascular development and homeostasis.^5^ In retinal capillaries, this is also reflected by the high prevalence of pericytes in the blood–retinal barrier (BRB) in comparison with other organs, as the ratio of pericyte to endothelial cell is 1:1–1:3.^4–9^ Pericytes of the iBRB and BBB are part of the neurovascular unit, together with endothelial cells, glial cells, microglia, neurons, and a basal lamina (Fig. 1).^10–12^ These cell types are in constant communication to regulate the permeability and function of the barrier in the brain and retina,^13^ thereby tightly controlling the transport of molecules and cells in and out of surrounding tissues. This highly specialized environment ensures proper functioning of neuronal tissue in the retina and brain.^14,15^

Under physiological conditions, pericytes maintain the barrier physically by regulating the polarization of astrocyte endfeet,^16^ by inhibiting transcytosis, and by inducing expression of tight junction proteins by endothelial cells.^14^ Pericytes locally regulate blood flow, clear harmful metabolites such as lactate released in the extracellular matrix by endothelial cells,^6,17^ control endothelial cell proliferation, and contribute to the basal lamina by secreting extracellular matrix proteins.^18,19^ In this way, pericytes stabilize existing and newly formed blood vessels.

To date, there is no single universal marker exclusively specific for pericytes in the iBRB and BBB. Pericytes are a heterogeneous cell population, and their identification often relies on a combination of markers. Markers that are often used in such a combination are platelet-derived growth factor receptor beta (PDGFRβ), nerve/glial antigen 2 (NG2), CD13 (also known as aminopeptidase N), alpha smooth muscle actin (αSMA), and desmin.^5,20,21^ A more recently identified pericyte marker is reduced folate carrier 1 (RFC1), also named solute carrier family 19 member 1 (SLC19A1).^22,23^ However, all these markers identify either a subset of pericytes in the tissue or are not uniquely expressed by pericytes, but by other cell types as well. Pericytes can be best identified on the basis of a combination of their location in capillary walls, morphological characteristics, and expression of molecular markers. Notably, there are differences in the ontogeny of pericytes in the central nervous system as compared with pericytes elsewhere in the body.^5^ Therefore, the marker expression of pericytes differs between organs, and there are also differences between species.^24–26^

To investigate the expression of various molecular markers in human retinal pericytes, we performed IF staining using antibodies against NG2, PDGFRβ, αSMA, CD13, and RFC1 in human retinal flatmounts. The NG2 proteoglycan, also known as chondroitin sulfate proteoglycan-4, melanoma proteoglycan, or high-molecular weight melanoma-associated antigen,^27^ is a transmembrane receptor that interacts extracellularly with more than 40 presumed ligands and can interact intracellularly with the cytoskeleton.^28,29^ NG2 is important in pericyte proliferation, motility, and formation and maturation of endothelial junctions through the integrin signaling pathway.^27,28,30^ It has been reported to have higher expression in activated pericytes in tumor stroma and wound healing tissues.^27^ PDGFRβ is a membrane-bound receptor, of which one of its major ligands, PDGF-B, is expressed by endothelial cells.^31,32^ Pericyte proliferation and recruitment to vascular endothelial cells are promoted by the PDGF-B/PDGFRβ signaling pathway.^33–36^ αSMA is a contractile protein found in smooth muscle cells and a subset of pericytes.^37,38^ Pericytes expressing αSMA were found at the branching points of retinal capillaries in mice, suggesting their role in vessel contraction.^38^ CD13 is a membrane-bound metalloprotease that proteolytically activates or inactivates regulatory peptides, such as enkephalins, neurotensin, and somatostatin.^18,19,39,40^ In addition, CD13 is involved in the nociception pathway and in inflammation through inactivation of the cytokine interleukin-8.^40^ CD13 promotes pericyte proliferation and migration in response to stimulation with angiogenic growth factors.^41,42^ In a previous in vitro study, increased expression of CD13 in migratory pericytes was observed, suggesting that the motility of pericytes may be increased, at least partly, via CD13 proteolysis.^42^ The novel pericyte marker RFC1, identified by single-cell expression profiling of retinal microvessels in adult mice,^22,23^ is a membrane transport protein. RFC1 is responsible for transport of the B9 family of vitamins, known as folates, or a subset of cyclic dinucleotides, driven by the export of organic anions across the BRB.^22,23^ The presence of RFC1 highlights the importance of pericytes in vitamin metabolism, which is crucial for DNA replication and repair.

The expression of these markers in pericytes may change in pathological conditions. Although pericytes in healthy tissue exhibit αSMA expression to a limited extent, αSMA expression alters in pericytes in DR, tumor angiogenesis, fibrosis, and inflammation.^5,43,44^ In DR, signaling through PDGFRβ is impaired, which may play a role in pericyte apoptosis.^5^ A reduction in NG2 expression in retinal pericytes was found in previous studies in streptozotocin-induced diabetic mice.^45–47^

IF staining provides spatiotemporal information about pericyte markers at a quantitative level.^48^ IF methodology optimization is key for each pericyte marker before studying the changes in marker expression in pericytes in pathological conditions. The current study includes retinal flatmounts instead of retinal sections to enhance spatial information. In this way, pericytes can be reconstructed in 3D after IF staining, and flatmounts provide insights in the pericyte location in relation to arteries, microvascular branching, and veins. To our knowledge, IF staining has not been performed on human retinal flatmounts for any pericyte marker apart from one study that investigated PDGFRβ in Alzheimer’s disease.^49^ Therefore, we have developed an IF protocol for detecting various pericyte markers in human retinal flatmounts. This IF methodology can be applied to study how various diseases affect pericytes in the human retina.

## Materials and Methods

### Human Retina

Human post-mortem eyes were provided by the Corneabank, Beverwijk, the Netherlands. The current research was performed in accordance with all requirements stated in the Dutch law “Wet op orgaandonatie” that describes the use of donor material for research purposes. According to this law, donors provide written informed consent for donation with an opt out of left-over material for related scientific research purposes. Specific requirements for the use for scientific research of left-over material originating from corneal grafting have been described in an additional document formulated by the Ministry of Health, Welfare, and Sport and the Bio Implant Services (BIS) Foundation (Eurotransplant; Leiden, the Netherlands, July 21, 1995; 6714.ht). The eyes were stored anonymously and, therefore, approval of their use by the Ethics Committee was not required by Dutch law. The use of human material was also in accordance with the Declaration of Helsinki on the use of human material for scientific research. Several hours post mortem, eyes were enucleated and the anterior parts of the eye, including the cornea and lens, were dissected. Fundoscopy images of the retina were taken before storage in the freezer and were evaluated by two independent ophthalmologists to determine the ophthalmological status. The eyecup was filled with Tissue-tek (cat# 4583, Sakura, Finetek Europe, Alphen aan de Rijn, the Netherlands) and eyes were snap frozen before being stored at −80C. Information on the type and duration of diabetes, when available, was kindly provided by the BIS Foundation. A summary of donor characteristics is listed in Table 1. Radial cuts of the eyecup were made to facilitate staining and microscopical imaging of the staining.

**Table 1.** Donor Information for Retinal Tissues.

Case	Ophthalmological Status	Sex	Age at Death (years)	Post-Mortem Delay (hr)	Diagnosis	Cause of Death
1	Normal	Male	49	15	Melanoma	Heart dysfunction
2	Normal	Female	54	20	Tendinitis wrist	Circulatory system complication
3	Normal	Female	68	14	Stomach cancer	Cancer
4	Normal	Female	67	5	Atherosclerosis, chronic kidney failure, percutaneous transluminal coronary angioplasty	Circulatory system complication

### Fixation of Retinal Tissue

The eyecup pieces were fixed with 4% formaldehyde (28908, Thermo Fisher Scientific, Bleiswijk, the Netherlands) at 4C and were washed twice in phosphate-buffered saline (PBS) for 10 min, after which the retina was detached from the eyecup and cut into sections to compare various experimental conditions. The sectioned retinas were collected in a polymerase chain reaction tube. Various additional fixation methods of human retinal flatmounts were tested: (1) methanol gradient fixation, which exists of a gradient of methanol in PBS with 0.1% Tween20 (PBST) for 10 min (25% > 50% > 75% > 100%), followed by rehydration with a reversed gradient (0.2 M hydrogen chloride in 100% methanol for 30 min > 75% (in PBST for 10 min) > 50% > 25%) at room temperature (RT); (2) cold methanol fixation with 100% methanol pre-chilled at −20C and incubated at −20C; or (3) no methanol fixation. After methanol treatment, flatmounts were washed twice in PBS for 10 min. To minimize the use of human tissue, 10-µm retinal cryosections were included for double staining with antibodies against PDGFRβ and RFC1, instead of flatmounts. Cryosections were air-dried for 20 min and fixed with 4% formaldehyde for 20 min. Sections were washed three times in PBS for 10 min. For all fixation conditions, the washing step was directly followed by incubation in blocking buffer.

### Permeabilization and Blocking of Retinal Tissue

Flatmounts and cryosections were subsequently incubated in blocking buffers for 1 hr at RT unless stated otherwise. Different blocking buffers, with various quantities of Triton X-100 (TX; T8787, Merck Life Science, Amsterdam, the Netherlands), fetal bovine serum (FBS; F7524, Merck Life Science), normal goat serum (NGS; 0060-01, Southern Biotech, Birmingham, AL), normal donkey serum (NDS; 0030-01, Southern Biotech), Tween20 (P1379, Merck Life Science), bovine serum albumin (BSA; 10735094001, Roche Diagnostics GmbH, Mannheim, Germany), or sodium azide (1.06688.0100, Merck Life Science) in PBS or Tris-buffered saline, were tested. Details on blocking buffer content are shown in Table 2. Blocking buffer 2 was previously described in the IF staining protocol for murine retinal wholemounts by van der Wijk et al.^50^ Immediately after the blocking procedure, flatmounts and cryosections were incubated with primary antibodies.

**Table 2.** Blocking Buffers Used in This Study.

Blocking Buffer Number	Blocking Buffer Content
1	PBS-0.3% TX + 0.2% BSA + 5% normal goat/donkey serum
2	PBS-3% TX + 1% FBS + 0.5% Tween20 + 0.1% sodium azide
3	PBS-0.1% TX + 10% normal donkey serum
4	PBS-0.1% TX + 5% BSA
5	PBS-0.1% TX + 2% BSA
6	PBS + 5% BSA
7	PBS-0.5% TX + 2% BSA + 0.2 M glycine
8	PBS-0.5% TX + 10% normal donkey serum
9	Tris-buffered saline-0.3% TX
10	PBS + 10% normal donkey serum

Abbreviations: BSA, bovine serum albumin; FBS, fetal bovine serum; PBS, phosphate-buffered saline; PBS-0.3% TX, PBS supplemented with 0.3% Triton X-100.

### Jasplakinolide Treatment for Actin Stabilization

For double staining of PDGFRβ and αSMA, fixation and blocking were adapted from the methods described in the study of Mai-Morente et al.^51^ Jasplakinolide was included as a F-actin stabilizing reagent to enhance αSMA detection. Flatmounts were first incubated with a blocking buffer. Immediately after blocking, flatmounts were incubated with jasplakinolide (20 µM; ab141409, Abcam, Amsterdam, the Netherlands) for 40 min at 4C, followed by fixation. Subsequently, flatmounts were washed three times for 10 min with PBS-0.05% TX. Alternatively, flatmounts were incubated with jasplakinolide followed by fixation and incubation with a blocking buffer. Then, flatmounts were washed three times for 10 min with PBS-0.05% TX. In addition, a condition without jasplakinolide was included.

### IF Staining of Pericyte Markers in Human Retinal Flatmounts and Cryosections

Flatmounts and cryosections were incubated overnight with primary antibodies against NG2, PDGFRβ, αSMA, CD13, and RFC1 at 4C (Table 3). Antibody solvents with various quantities of TX, FBS, NGS, NDS, Tween20, BSA, or sodium azide in PBS or Tris-buffered saline were tested and are described in Table 4. Antibody solvent 4 was previously described in the IF staining protocol for hippocampal slices by Mai-Morente et al.^47^ In addition, commercially available (phosphate-buffered) normal antibody diluent (ABB999, ScyTek Laboratories, Logan, UT) was tested as antibody solvent as well as antibody solvents identical to the blocking buffer (NG2; CD13).

**Table 3.** Primary Antibodies Used in This Study.

Antigen	Host Species	Source	Working Dilution	Working Concentration
Collagen type IV	Mouse	Invitrogen, MA1-22148	1:1000	7.9 µg/ml
Laminin	Rabbit	Abcam, Ab11575	1:1000	0.72 µg/ml
NG2	Mouse	Merck Life Science, MAB2029	1:100	10 µg/ml
PDGFRβ	Goat	R&D Systems, AF385	1:100	2 µg/ml
αSMA	Mouse	DAKO, M0851	1:250–1000	71–284 µg/l
CD13	Mouse	R&D Systems, MAB3815	1:200	2.5 µg/ml
RFC1	Rabbit	Merck Life Science, AV44167	1:100–500	1–10 µg/ml

Invitrogen (Waltcam, MA); R&D Systems (Minneapolis, MI); DAKO (Santa Clara, CA).

**Table 4.** Solvents Used for Antibody Dilution in This Study.

Solvent Number	Solvent Content
1	(Phosphate-buffered) normal antibody diluent
2	PBS-0.01% TX + 1% BSA
3	PBS-0.01% TX + 0.2% BSA
4	PBS-0.5% TX + 2% BSA

Different antibody concentrations were tested for RFC1 and αSMA (Table 3). As a negative control, primary antibodies were omitted. After primary antibody incubation, flatmounts or sections were washed three times with PBS-0.3% TX for 20 min (NG2; PDGFRβ), or three times with PBS for 20 min (PDGFRΒ) or 10 min (PDGFRβ and RFC1), or six times with PBS for 10 min (PDGFRβ; PDGFRβ and NG2; PDGFRβ and CD13), or two times with PBS-0.05% TX for 10 min (PDGFRβ and αSMA). Flatmounts or sections were incubated with secondary antibodies (Table 5) for 2 hr at RT in the dark in the same antibody solvent as was used for primary antibodies. Following secondary antibody incubation, flatmounts were washed in the same manner as after primary antibody incubation, apart from double staining of PDGFRβ and αSMA, that was washed three times with PBS-0.05% TX for 10 min. Retinal sections were washed three times with Tris-buffered saline for 5 min or three times with PBS for 10 min.

**Table 5.** Secondary Antibodies Used in This Study.

Antibody	Conjugate	Source	Working Dilution	Working Concentration
Donkey anti-rabbit IgG	Alexa Fluor 488	Invitrogen, A-21206	1:400	5 µg/ml
Donkey anti-mouse IgG	Alexa Fluor Plus 488	Invitrogen, A32766	1:1000	2 µg/ml
Goat anti-mouse IgG	Cy3	Jackson, 115-165-166	1:100	15 µg/ml
Goat anti-rabbit IgG	Cy3	Jackson, 111-165-144	1:400	3.75 µg/ml
Donkey anti-goat IgG	Cy3	Jackson, 705-165-147	1:200	7.5 µg/ml
Goat anti-mouse IgG	Alexa Fluor 633	Invitrogen, A-21052	1:500	4 µg/ml
Goat anti-rabbit IgG	Alexa Fluor 633	Invitrogen, A-21071	1:200	10 µg/ml
Donkey anti-rabbit IgG	Alexa Fluor Plus 647	Invitrogen, A32795	1:1000	2 µg/ml
Donkey anti-mouse IgG	Alexa Fluor 647	Invitrogen, A31571	1:200	10 µg/ml

Jackson ImmunoResearch Laboratories (West Grove, PA).

### Mounting of Retinal Tissue

After the final washing step, flatmounts were positioned on a microscope slide with the inside of the eye facing upward. Flatmounts or cryosections were mounted with Vectashield antifading mounting medium containing 4′,6-diamidino-2-phenylindole (DAPI; H-1200-10, Vector Laboratories, Newark, CA), covered with a cover glass and sealed with transparent nail varnish.

### Microscopical Analysis of Pericyte Staining

Flatmounts and sections were imaged using a Leica STELLARIS confocal microscope (Leica Microsystems, Wetzlar, Germany) with a HC APO CS2 40×/1.30 oil-immersion objective. The confocal images were made with bidirectional sequential scanning at 1024 × 1024 resolution with a scanning speed of 600 Hz and line average of 8. Acquired images were processed and exported from Leica Application Suite X.

### Expression Analysis of CD13 and RFC1

The mRNA expression of ANPEP (CD13) and SLC19A1 (RFC1) in pericytes of the mature human eye, specifically in the retina, was explored using the following resources: The Human Protein Atlas (http://www.proteinatlas.org/, accessed on December 16, 2024), the Human Eye Transcriptome Atlas v3.0 (https://www.eye-transcriptome.com/index.php, accessed on December 17, 2024), and the Eye Integration database of the National Eye Institute (https://eyeintegration.nei.nih.gov/, v2.12, accessed on December 17, 2024). This analysis utilized RNA sequencing data generated by the Genotype-Tissue Expression project accessed from Tissue data in the Human Protein Atlas, selecting “Retina” as tissue. In the Human Eye Transcriptome Atlas, mRNA data of ANPEP and SLC19A1 in the “Retina periphery” and “Retina center” were selected. mRNA expression of ANPEP and SLC19A1 reported by eyeIntegration was accessed by the Single Cell Plots function, selecting “Mature” and “Pericyte” for each gene. mRNA data from the Human Protein Atlas were analyzed using GraphPad Prism 10 (GraphPad Software, Inc., La Jolla, CA). A Mann–Whitney *U* test was performed to compare the expression of each gene between human and mouse data. Statistical significance was determined as *p*<0.05.

## Results

Human retinal flatmounts were immunofluorescently stained to localize pericytes using antibodies against NG2, PDGFRβ, αSMA, CD13, and RFC1.

### NG2 Staining

**Figure 2.**
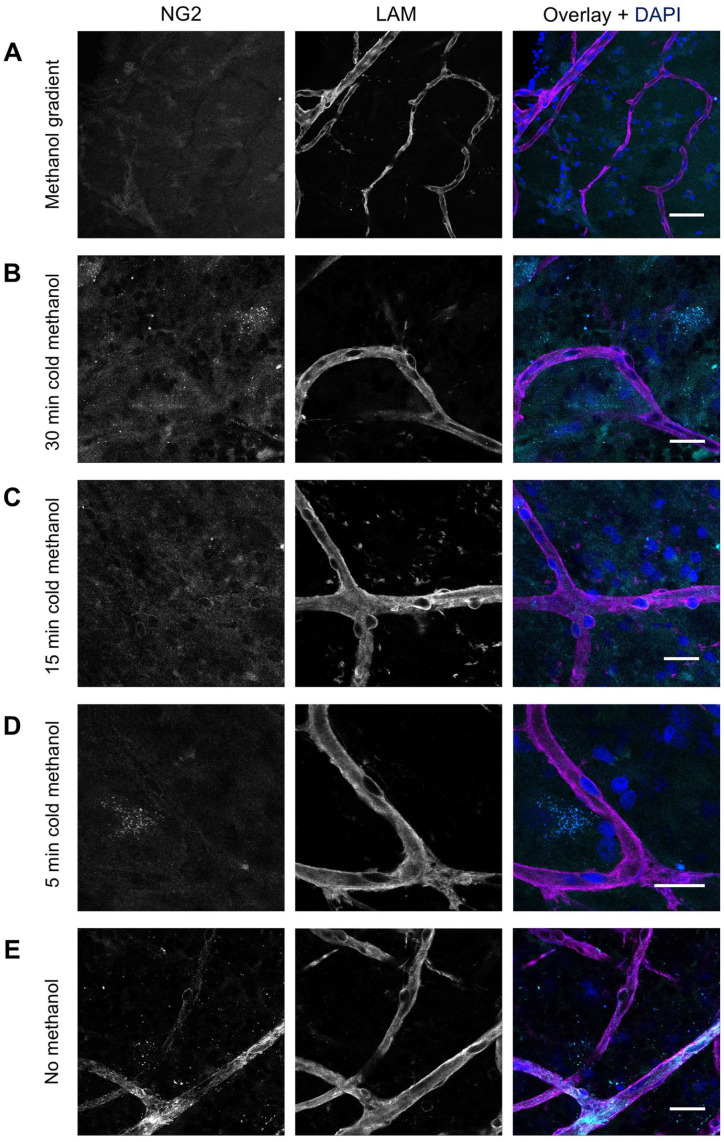
Effect of different fixation methods on immunofluorescence staining of NG2 in human retinal flatmounts. Flatmounts were stained for laminin (LAM) for basal lamina (magenta), NG2 for pericytes (cyan) and DAPI for nuclei (blue) after different fixation methods. After initial fixation with 4% formaldehyde, additional fixation was performed with (A) a methanol gradient, (B) cold 100% methanol for 30 min, (C) cold 100% methanol for 15 min, (D) cold 100% methanol for 5 min, or (E) no methanol. (Scale bars A–E = 25 μm.)

IF staining with anti-NG2 antibodies was developed to detect NG2^+^ pericytes in the human retina. Various fixation methods were tested to compare staining results using anti-NG2 antibodies, including fixation with formaldehyde followed by (1) fixation with a methanol gradient; (2) cold 100% methanol (−20C) for 5, 15, or 30 min; or (3) no methanol fixation. Methanol can improve tissue penetration and may help unmask epitopes that are masked by formaldehyde crosslinking, but on the other hand, it may disrupt established crosslinks. NG2-antibody signal was not detectable after fixation conditions 1 and 2, which both included methanol (Fig. 2A to D). Only condition 3, without the use of methanol, resulted in staining of pericytes.

**Figure 3.**
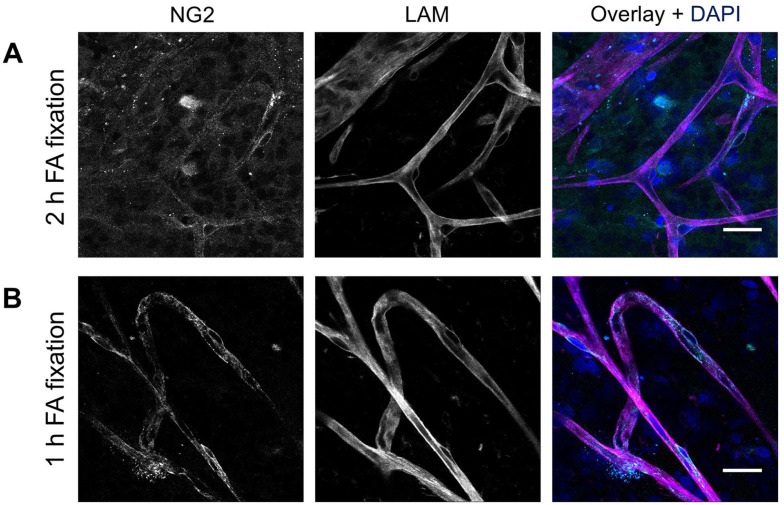
Effect of fixation time on immunofluorescence staining of NG2 in human retinal flatmounts. NG2 staining (cyan) after formaldehyde fixation for 2 hr (A) or 1 hr (B). Basal lamina was stained with anti-laminin (LAM) antibodies (magenta) and nuclei with DAPI (blue). (Scale bars A and B = 25 μm.)

Next, the impact of formaldehyde fixation time on NG2 staining was studied. Retinal flatmounts fixed for 1 hr exhibited stronger and more specific NG2 staining of pericytes as compared with 2 hr fixation (Fig. 3), indicating that shorter fixation time better preserves the epitopes necessary for optimal antibody binding.

**Figure 4.**
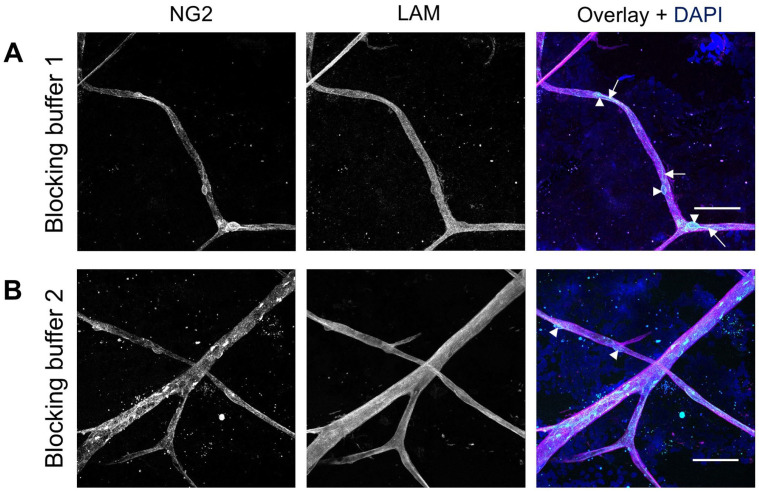
Effect of different blocking buffers on immunofluorescence staining of NG2 in human retinal flatmounts. Specific NG2 staining (cyan) was observed after incubation with blocking buffer 1 (A) and blocking buffer 2 (B). Basal lamina was stained with anti-laminin (LAM) antibodies (magenta) and nuclei with DAPI (blue). Arrowheads indicate pericyte cell bodies and arrows indicate pericyte processes. (Scale bars A and B = 50 μm.)

The composition of the blocking buffer was also optimized to enhance specific NG2 staining. Of the two blocking buffers used, blocking buffer 1 (Table 2, Fig. 4A) and blocking buffer 2 (Fig. 4B), blocking buffer 1 provided the best NG2 staining. This buffer minimized nonspecific staining and allowed for clearer visualization of both the pericyte cell bodies and their processes in the human retina. Optimal staining conditions for NG2 are listed in Table 6.

**Table 6.** Optimal Conditions for IF Staining of Pericytes in Human Retinal Flatmounts.

Markers	Fixation	Blocking Buffer	Antibody Solvent	Optimal Dilution Primary Antibody	Wash Solution
NG2	4% FA for 1 hr	PBS-0.3% TX + 0.2% BSA + 5% normal serum	PBS-0.3% TX + 0.2% BSA + 5% normal serum	NG2 1:100	PBS-0.3% TX
NG2 + PDGFRβ	4% FA for 1 hr	PBS-0.3% TX + 0.2% BSA + 5% normal serum PBS-0.1% TX + 2% BSA	PBS-0.01% TX + 0.2% BSA	NG2 1:100, PDGFRβ 1:100	PBS
αSMA + PDGFRβ	100% methanol for 20 min	PBS-0.5% TX + 2% BSA + 0.2M glycine	PBS-0.01% TX + 2% BSA	αSMA 1:500, PDGFRβ 1:100	PBS-0.05% TX

### PDGFRβ Staining

Our study aimed to perform a double staining with antibodies for PDGFRβ and NG2 to assess simultaneous marker expression in pericytes and potentially identify pericyte subsets. In the initial trials for optimizing PDGFRβ staining, NG2 antibodies were excluded to minimize antibody usage.

**Figure 5.**
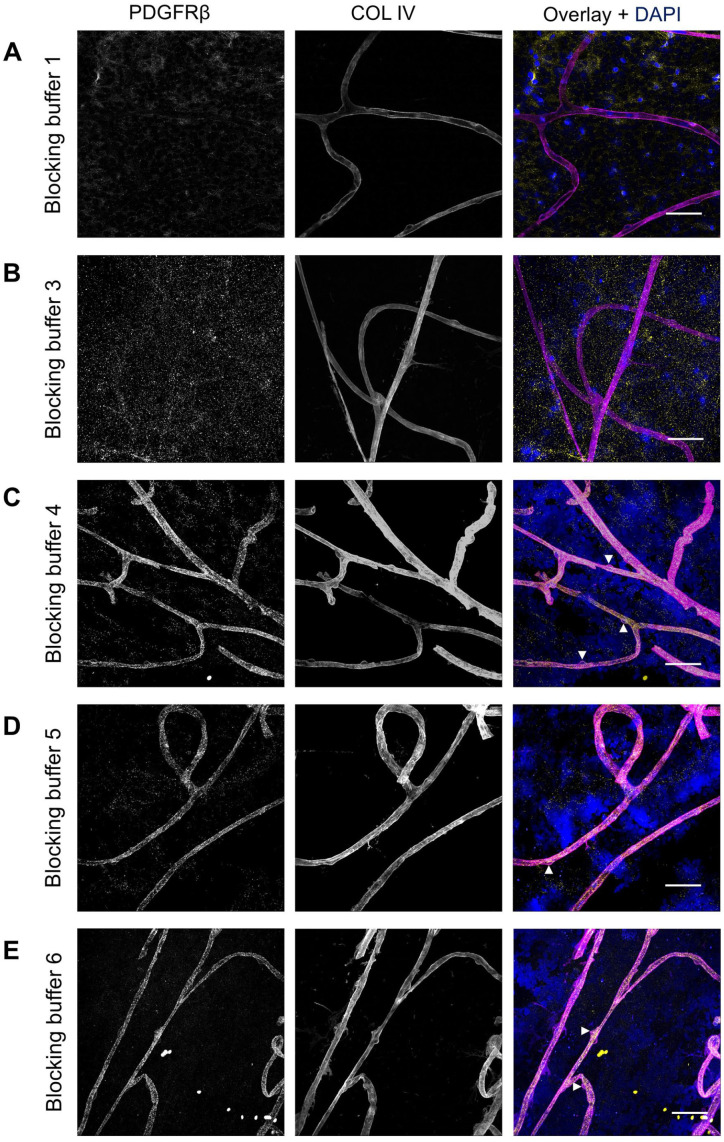
Effect of different blocking buffers on immunofluorescence staining of PDGFRβ in human retinal flatmounts. The pericyte marker PDGFRβ (yellow) was immunostained using antibodies in conditions optimized for NG2 staining, performed with blocking buffer 1 (A) or buffer 3–6 (B-E). Basal lamina was stained with anticollagen type IV (COL IV) antibodies (magenta) and nuclei with DAPI (blue). Examples of pericytes are indicated with arrowheads. (Scale bars A–E = 50 μm.)

PDGFRβ staining on human retinal flatmounts was initially tested using the same protocol established for NG2 staining. However, these conditions did not result in positive staining of PDGFRβ (Fig. 5A). Altering blocking buffer 1 to blocking buffer 3, thereby reducing the percentage of TX and replacing BSA by an increased percentage of normal serum (Table 2), also failed to improve PDGFRβ staining (Fig. 5B). Specific staining of PDGFRβ was successfully achieved after applying blocking buffer 4, 5, or 6 (Fig. 5C to E), that all contained low amounts of TX or no TX and in which normal serum was replaced by BSA. Optimal results were obtained after blocking with blocking buffer 5 (Fig. 5D). Apparently, the omission of normal serum from the blocking buffer improved the specificity of PDGFRβ staining, suggesting that the absence of normal serum may reduce nonspecific binding and enhance the immunodetection of PDGFRβ^+^ pericytes.

**Figure 6.**
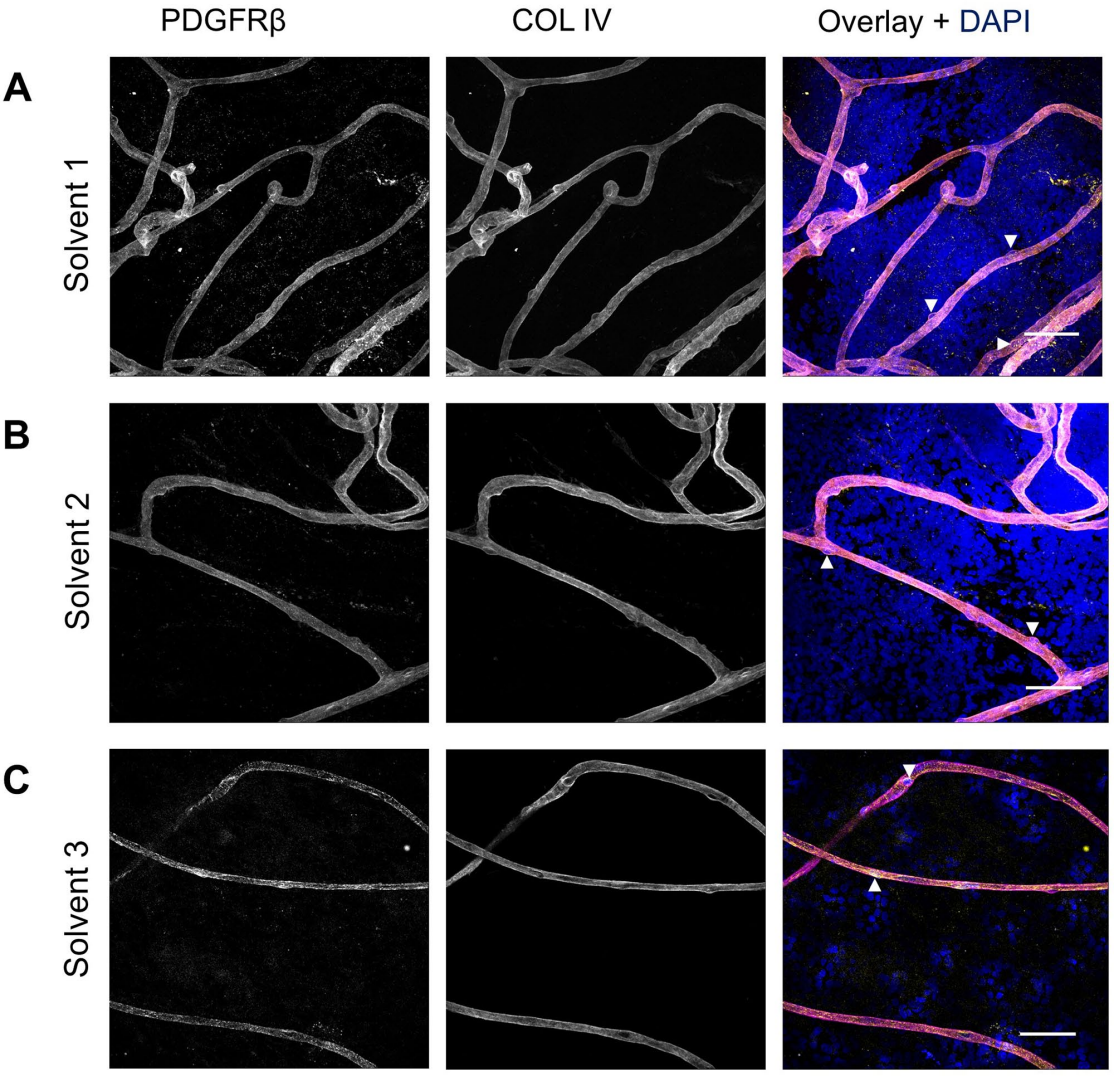
Effect of different antibody solvents on immunofluorescence staining of PDGFRβ in human retinal flatmounts. Specific PDGFRβ staining (yellow) was obtained when using antibody solvent 1 (A), solvent 2 (B), or solvent 3 (C). Basal lamina was stained with anticollagen type IV (COL IV) antibodies (magenta) and nuclei with DAPI (blue). Examples of pericytes are indicated with arrowheads. (Scale bars A–C = 50 μm.)

In addition to various blocking buffers, different antibody solvents (Table 4) were compared: solvent 1 (Fig. 6A), solvent 2 (Fig. 6B), and solvent 3 (Fig. 6C). All experimental conditions incorporated the application of blocking buffer 5. Although all three conditions resulted in specific PDGFRβ staining, the use of solvent 1 produced the lowest background signal, providing the clearest and most specific staining (Fig. 6A). This suggests that the normal antibody diluent was the most effective for minimizing nonspecific binding and improving the overall signal-to-noise ratio for PDGFRβ detection.

### NG2 and PDGFRβ Double Staining

**Figure 7.**
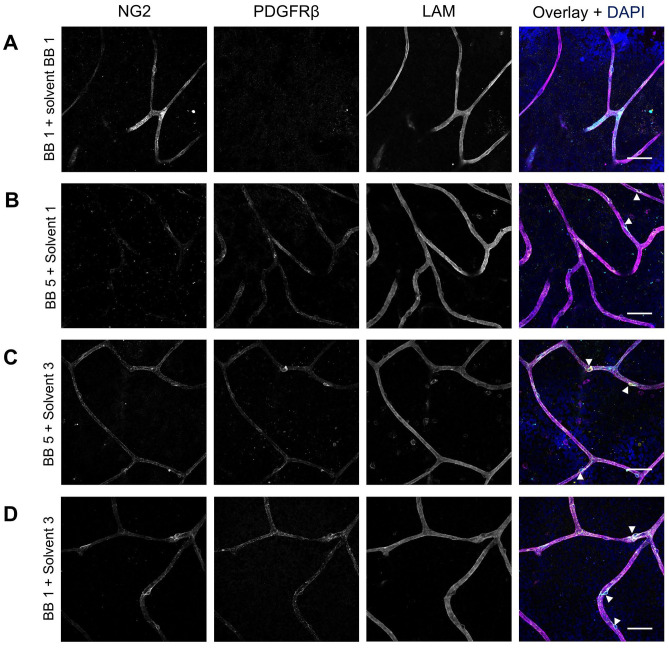
Effect of different blocking buffers and antibody solvents on immunofluorescence double staining of NG2 and PDGFRβ in human retinal flatmounts. The immunofluorescence conditions optimized for NG2 (A) or PDGFRβ (B) did not result in specific double staining of NG2 (cyan) and PDGFRβ (yellow). Optimized immunofluorescence conditions for PDGFRβ were tested with solvent 3 as antibody solvent instead of solvent 1 (C) and optimized immunofluorescence conditions for NG2 were tested with solvent 3 as antibody solvent instead of blocking buffer 1 as antibody solvent (D). Basal lamina was stained with anti-laminin (LAM) antibodies (magenta) and nuclei with DAPI (blue). Examples of pericytes are indicated with arrowheads. (Scale bars A–D = 50 μm.)

**Appendix Figure 1.**
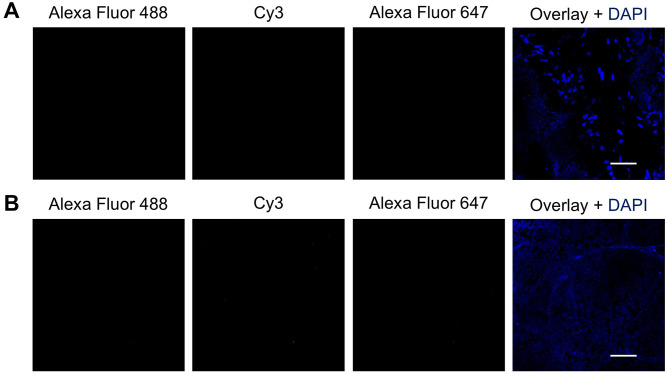
Representative immunofluorescence staining images of negative controls of human retinal flatmounts. Immunostaining with secondary antibody only as a negative control under identical conditions for staining of (A) NG2, PDGFRβ, and laminin and (B) αSMA, PDGFRβ, and laminin. Nuclei were stained with DAPI (blue). (Scale bars A and B = 50 μm.)

After separately optimizing the staining of NG2 and PDGFRβ, the protocol for double staining of these markers was optimized. As shown in Fig. 7A, the optimized condition for NG2 staining did not result in positive PDGFRβ staining, and similarly, Fig. 7B demonstrates that the optimized PDGFRβ conditions did not yield positive NG2 staining. However, when the optimized IF conditions for PDGFRβ were applied with solvent 3 (10× diluted blocking buffer 5) as antibody solvent, instead of solvent 1, the retinal flatmounts showed specific expression of both NG2 and PDGFRβ (Fig. 7C). Likewise, when the optimized IF staining condition for NG2 was applied with solvent 3 instead of blocking buffer 1 as antibody solvent, strong expression of both NG2 and PDGFRβ was observed in retinal flatmounts (Fig. 7D). Negative control flatmounts revealed no unspecific staining caused by the application of the secondary antibodies (Appendix Fig. 1). These results suggest that optimizing the antibody solvent composition is crucial for achieving specific and robust double staining of NG2 and PDGFRβ, enabling the detection of both markers simultaneously in retinal tissue. Optimal staining conditions for NG2 and PDGFRβ double staining are listed in Table 6 and the result is shown at higher magnification in Fig. 9A.

### αSMA and PDGFRβ Double Staining

For double staining with αSMA and PDGFRβ antibodies, we adapted the IF staining protocol for hippocampal slices of Mai-Morente et al.^51^ The protocol includes the use of jasplakinolide, a reagent that stabilizes F-actin by promoting actin filament polymerization, which is essential to detect αSMA in pericytes using IF staining. To achieve this, flatmounts were incubated with jasplakinolide before fixation.

**Figure 8.**
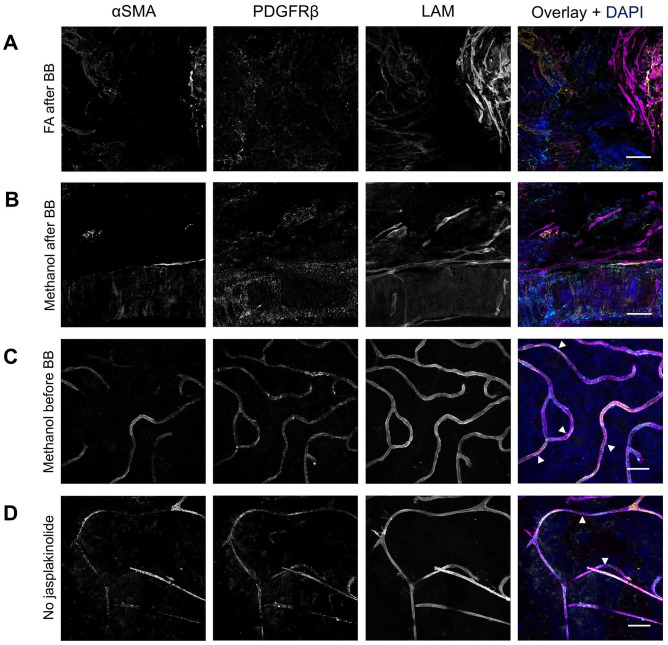
Effect of different fixatives and jasplakinolide on immunofluorescence double staining of αSMA and PDGFRβ in human retinal flatmounts. Immunofluorescence staining of αSMA (yellow) and PDGFRβ (cyan) after fixation with 4% formaldehyde (A) or methanol (B and C). Tissue integrity was compromised after fixation with formaldehyde (A) and fixation with methanol (B) after blocking buffer incubation. αSMA and PDGFRβ staining was best after fixation with methanol before application of the blocking buffer in combination with the use of jasplakinolide (C). αSMA and PDGFRβ staining without the use of jasplakinolide (D). Basal lamina was stained with anti-laminin (LAM) antibodies (magenta) and nuclei with DAPI (blue). Examples of pericytes are indicated with arrowheads. (Scale bars A–D = 50 μm.)

When fixation of retinal flatmounts with 4% formaldehyde for 20 min (Fig. 8A) or 100% methanol for 20 min (Fig. 8B) was performed after incubation with blocking buffer 7 for 2 hr at RT, the tissue morphology was severely damaged. To preserve tissue integrity, the fixation step with methanol was performed before incubation with blocking buffer rather than afterward (Fig. 8C). As a result, αSMA^+^ PDGFRβ^+^ pericytes were clearly visible in the human retinal flatmounts. When incubation with jasplakinolide was omitted, specific αSMA was still detected (Fig. 8D). However, there was an increase in nonspecific signal, and the integrity of the flatmounts was compromised. Negative control flatmounts revealed no unspecific staining caused by the application of the secondary antibodies (Appendix Fig. 1).

**Figure 9.**
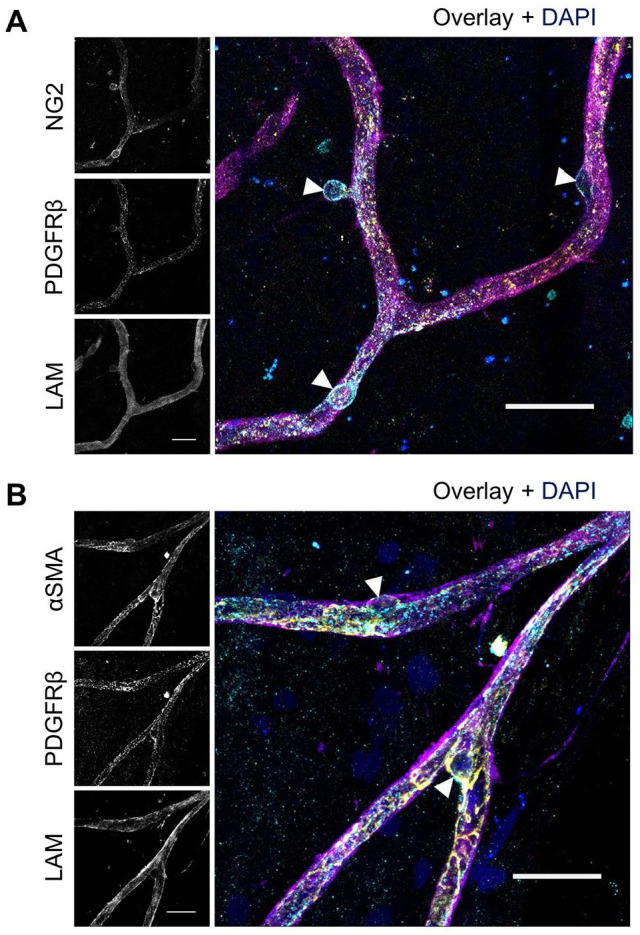
Immunofluorescence double staining of NG2 and PDGFRβ and of αSMA and PDGFRβ in human retinal flatmounts. Higher magnification images of pericytes detected with immunofluorescence double staining of (A) NG2 (cyan) with PDGFRβ (yellow) and (B) αSMA (yellow) with PDGFRβ (cyan). Pericyte bodies are indicated with arrowheads. Basal lamina was stained with anti-laminin (LAM) antibodies (magenta) and nuclei with DAPI (blue). (Scale bars A and B = 25 μm.)

These results suggest that the use of jasplakinolide is crucial for achieving specific staining of αSMA. The order of incubation with jasplakinolide followed by fixation and blocking enables the detection of both αSMA and PDGFRβ simultaneously in retinal tissue. Optimal staining conditions for αSMA and PDGFRβ double staining are listed in Table 6 and the result is shown at higher magnification in Fig. 9B.

### CD13 and PDGFRβ Double Staining

**Figure 10.**
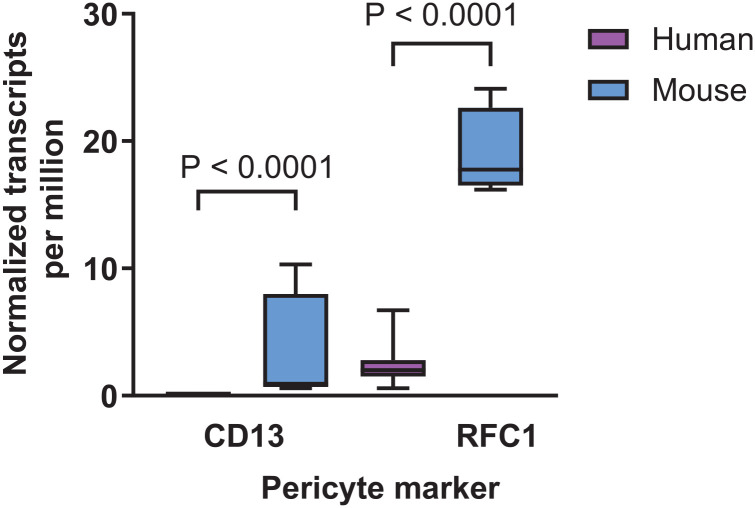
CD13 and RFC1 mRNA expression levels in the human and mouse retina. RNA sequencing data for CD13 and RFC1 are shown in boxplots as normalized transcripts per million for human retina (magenta bars) and mouse retina (blue bars). Box plots indicate median, min, and max and first and third quartile. Based on GTEx RNA-seq data from the Human Protein Atlas.

**Appendix Figure 2.**
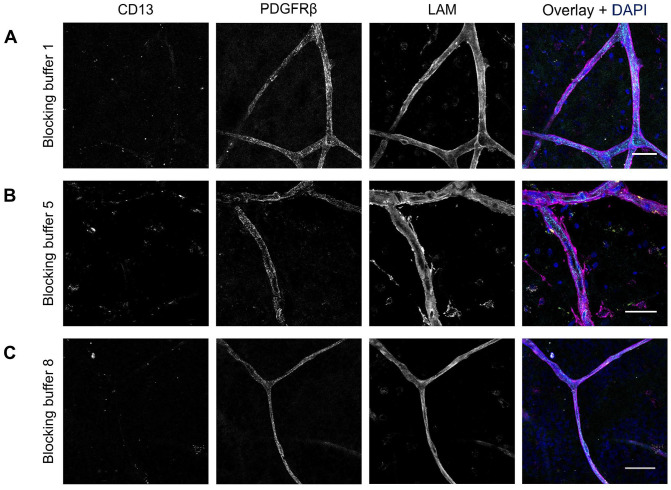
Effect of different blocking buffers on immunofluorescence double staining of CD13 and PDGFRβ in human retinal flatmounts. The pericyte markers CD13 (yellow) and PDGFRβ (cyan) were immunostained using antibodies in conditions optimized for double staining of NG2 and PDGFRβ, performed with blocking buffer 1 (A), blocking buffer 5 (B), or blocking buffer 8 (C). Basal lamina was stained with anti-laminin (LAM) antibodies (magenta) and nuclei with DAPI (blue). (Scale bars A–C = 50 μm.)

**Appendix Figure 3.**
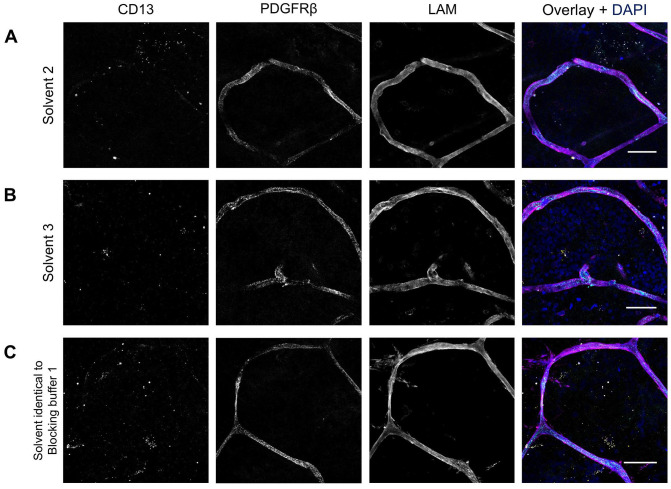
Effect of different antibody solvents on immunofluorescence double staining of CD13 and PDGFRβ in human retinal flatmounts. Immunofluorescence double staining of CD13 (yellow) and PDGFRβ (cyan) after testing antibody solvent 2 (A), solvent 3 (B), or a solvent identical to blocking buffer 1 (C). Basal lamina was stained with anti-laminin antibodies (magenta) and nuclei with DAPI (blue). (Scale bars A–C = 50 μm.)

Our study aimed to perform a double staining with antibodies for CD13 and PDGFRβ. As shown in Appendix Fig. 2A and B, the conditions optimized for NG2 and PDGFRβ were tested for this purpose, but did not result in positive CD13 staining. Altering the blocking buffer 1 and 5 to blocking buffer 8 also failed to improve CD13 staining (Appendix Fig. 2C). Furthermore, no specific signal for CD13 was detected when varying the antibody solvents (Appendix Fig. 3). Because no optimal staining condition could be identified for CD13 staining in human retinal flatmounts, and CD13 staining has only been reported in mouse retinas, we considered the possibility that CD13 expression may differ between species. According to RNA sequencing data from the Human Protein Atlas, CD13 expression is reported in the mouse retina (3.2 normalized transcripts per million), whereas CD13 expression in human retina is completely absent (Fig. 10). Similarly, the Human Eye Transcriptome Atlas and the Eye Integration database from the National Eye Institute found a low number of reads for CD13 in pericytes in the mature human eye. The absence of mRNA expression may well be an explanation for the failure to detect CD13 protein in the human retina.

### RFC1 and PDGFRβ Double Staining

**Appendix Figure 4.**
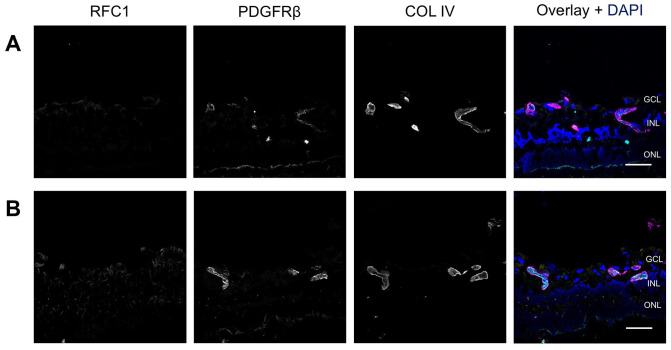
Comparing optimized immunofluorescence protocols for immunofluorescence double staining of RFC1 and PDGFRβ in human retinal cryosections. Immunofluorescence double staining of RFC1 (yellow) and PDGFRβ (cyan) using the optimized double staining protocol for PDGFRβ and NG2 (A) or using an adapted protocol from the study by Gurler et al.^22^ (B). Basal lamina was stained with anticollagen type IV (COL IV) antibodies (magenta) and nuclei with DAPI (blue). (Scale bars A and B = 50 μm.) Abbreviations: GCL, ganglion cell layer; INL, inner nuclear layer; ONL, outer nuclear layer.

For double staining of RFC1 and PDGFRβ, we first tested the conditions optimized previously for double staining of NG2 and PDGFRβ in retinal cryosections. Appendix Fig. 4A shows that these conditions resulted in positive staining of PDGFRβ, but not of RFC1. We also adapted and tested the IF staining protocol of Gurler et al.,^22^ performing permeabilization with blocking buffer 9 for 30 min at RT followed by incubation with blocking buffer 10 for 1 hr at RT. These staining conditions did not improve detection of RFC1 in the retinal vasculature (Appendix Fig. 4B). No optimal staining condition could be found for RFC1 staining in human retinal tissue. This was in line with the Human Protein Atlas for RNA sequencing data, which showed that RFC1 mRNA expression (2.2 normalized transcripts per million) in human retina is considerable lower than that in the mouse retina (Fig. 10). Similarly, the Human Eye Transcriptome Atlas and the Eye Integration database from the National Eye Institute found a low number of reads for RFC1 in pericytes in the mature human eye. The low mRNA expression may be an explanation for the lack of RFC1 protein detection in the human retina.

## Discussion

In this study, we developed IF staining protocols to detect NG2, αSMA, and PDGFRβ as pericyte markers in human retinal flatmounts. Based on IF staining of these pericyte markers, we were able to identify pericytes with typical morphological characteristics: pericytes that have a cell body with a prominent nucleus and large processes covering blood vessels. CD13 and RFC1 could not be detected in the human retina in contrast to their reported expression in mouse retina. We have identified variations in the IF staining protocol that led to the most significant improvement in staining for each marker. Appropriate fixation is essential for frozen tissues to improve tissue morphology and proper detection of the protein of interest.^52,53^ The importance of fixation solution and timing of fixation is illustrated by this study. For NG2 staining, fixation with methanol did not result in a positive staining signal. On the contrary, fixation with formaldehyde resulted in specific NG2 signal. This outcome is in contrast to earlier findings which have suggested that methanol is suitable to fix retinal wholemounts for immunostaining,^54^ also specifically for NG2 staining.^10,38,55^ This discrepancy could be attributed to the difference in species as murine retinas were used in previous studies. Also, the importance of timing of fixation was demonstrated here for the double staining with PDGFRβ and αSMA antibodies. Fixation enhances specific IF signal by preventing autolytic activity of enzymes in the tissue and conserves tissue morphology by crosslinking macromolecules.^56,57^ Delaying fixation leads to protein degradation in the retina, and thus reduces the IF signal as well as tissue morphology.

Selecting the appropriate detergents and blocking reagents is crucial to optimize IF staining. Detergents such as Tween20 or TX allow antibodies to access intracellular epitopes, but using too much of these agents may lead to loss of pericyte marker detection for proteins that are sensitive to detergents.^58^ Detergents in washing buffers help to remove nonspecifically bound antibodies, resulting in more specific staining. Excessive presence of detergents may also wash off specifically bound antibodies, resulting in less specific staining. Blocking reagents reduce nonspecific antibody binding and background noise by covering reactive sites on the antigens in the tissue and can additionally stabilize cellular morphology.^59^

We recommend to optimize the IF staining protocols for each tissue type and protein of interest. Parameters that can be adjusted to optimize specific staining, and that were not shown in this study, include: antibody concentration, antibody clonality, isotype and host species, temperature and duration of each incubation step, composition of washing solvent, number of washing steps, type of mounting medium, and the use of fresh tissue instead of frozen tissue.^60,61^ When using frozen tissue for IF staining, limiting post-mortem time improves protein signal detection in immunostaining.^62^ Antibody clonality affects IF staining because monoclonal antibodies are more specific, whereas polyclonal antibodies provide higher sensitivity. Matching antibody isotype and host species ensures compatibility between epitope, primary and secondary antibodies and preventing species-on-species nonspecific staining. Optimization of washing further improves specific staining, because the washing step allows for removal of antibodies that are bound nonspecifically. In addition, appropriate microscope settings and software settings for image acquisition and image analysis are essential to obtain high-quality and accurate images and help achieve a high signal-to-noise ratio while limiting photobleaching.^63^

After optimization of the IF staining, all markers were detected and localized in pericytes in both the cell body and the processes covering the blood vessel. We are the first to report a staining protocol for multiple pericyte markers in human retinal flatmounts. Using retinal flatmounts instead of retinal tissue sections has several advantages. First, flatmounts allow for visualization of the entire vascular network, which is not possible in tissue sections. This provides information on the location of the pericyte, for example regarding the proximity to an arteriole or venule or regarding the branch order of capillaries. Furthermore, pericytes that are migrating away from blood vessels can be visualized. In addition, visualization of pericytes in a 3D reconstruction image is easier in flatmounts compared with serial tissue sections.

Using our protocol, we observed that the expression of NG2 was evenly distributed throughout pericytes in the human retina. These results are consistent with the staining pattern previously found in human retina.^64^ In addition, the staining pattern found in humans was similar to that in retinas of mice^10,55,65,66^ and rats.^67^ In the current study, PDGFRβ was detected as granulated staining in pericytes. The granulated staining pattern found here matches with the staining pattern previously found in human retina^49^ and murine retina.^68^ PDGFRβ is primarily localized at the pericyte membrane and internalization of PDGFRβ upon ligand binding relocates PDGFRβ to endosomes that can attribute to the granulated appearance of PDGFRβ staining.^31,69^ Staining patterns in other studies that reported staining of PDGFRβ in murine retina could not be assessed properly due to the low magnifications used in these studies.^66,70^ For αSMA, we found a patchy appearance localizing at blood vessels of the human retina. This finding corresponds to the staining pattern found in human retina^43^ and mouse retina.^38,51,71^ The expression of each pericyte marker did not fully colocalize with that of the other pericyte markers. The observed differences in expression location and expression pattern of the different pericytes markers may be attributed to the distinct function of pericytes or state of maturity, suggesting the presence of pericyte subpopulations.^20,21,44,72–74^ For example, arteriolar pericytes express NG2 and α-SMA, whereas capillary pericytes lack these markers in some tissues. This distinction suggests that arteriolar pericytes may play role in blood flow regulation, linked to their contractile function. Postcapillary pericytes that lack NG2 have been shown to regulate neutrophil movement across the basal lamina in muscles.^75^ However, little is known about pericyte heterogeneity and the function of pericyte subpopulations.^20,73^ Therefore, double staining of pericyte markers is crucial to identify pericyte subpopulations.

Other reported pericyte markers include CD13,^76,77^ also known as aminopeptidase N, and SLC19A1,^22,23^ also known as RFC1. However, immunostaining of CD13 and RFC1 was only reported in the mouse retina and not yet in the human retina. IF staining of these markers was tested in the current study. The antibodies detecting human CD13 and RFC1 did not show any immunoreactivity in pericytes in the human retina. For CD13, our results are in line with RNA sequencing data from the Human Protein Atlas. Although for RFC1 mRNA expression was found in the human retina, its expression was significantly lower than that in mouse retina. The Human Eye Transcriptome Atlas and the Eye Integration database from the National Eye Institute reported a low number of reads for both CD13 and RFC1 in pericytes in the mature human eye. The presence of mRNA in the human retina may be due to expression in other cell types in the retina than pericytes, for example RFC1 is also expressed in retinal microglia.^78^ The difference in pericyte marker expression between mice and humans highlights the importance of established IF protocols specifically tailored for human tissues.

IF staining allows multiple pericyte markers and supporting markers to be simultaneously stained in flatmounts.^59^ In addition, visualization of pericytes by immunostaining provides information on pericyte number, morphology, and location, and permits quantitative microscopy.^59,67,68^ The limitation of using fluorophores to detect proteins of interest is the possibility of photobleaching of fluorophores and autofluorescence of the tissue of interest.^48,59^ To circumvent the issues related to fluorophores, chromogenic immunohistochemistry offers a staining alternative that also provides additional information on the tissue morphology.^59,79^ However, IF staining offers increased sensitivity, 3D visualization, quantification possibilities related to abundance of protein, and multiplexing opportunities compared with chromogenic immunohistochemistry.^59,80^ An alternative technique to visualize pericytes in human retina includes electron microscopy.^81,82^ Electron microscopic studies provide information on pericyte number and morphology, but functional information based on pericyte markers and numbers is lacking. Another drawback is that the thin sections used in electron microscopy offer limited spatial information in relation to the vasculature. Another limitation of IF staining and electron microscopy is the use of fixed, non-living cells that are used to seek information regarding the state in living organisms, tissues, and cells. Visualization of pericytes in the living human retina is possible by combining adaptive optics scanning laser ophthalmoscopy with a modified dark-field detection scheme.^83–85^ This technique provides information on pericyte number and location, and visualization in living retina allows for studying of pericytes during disease progression or during treatment. However, this technique cannot identify pericyte subsets based on pericyte markers.

Pericytes are widely spread throughout the human body and play an important role in vascular flow and homeostasis.^7,20,86^ Pericytes also have roles not related to the vasculature, depending on the location in different organs. The prevalence of pericytes in the retina is, together with the brain, significantly higher compared with other organs to enable proper functioning of the neuronal tissue.^4,5,7–9,87^ In the retina, pericytes share the basal lamina with endothelial cells and provide structural support by covering most of the microvascular area.^4,9,86,88,89^ During angiogenesis, pericytes regulate endothelial cell proliferation and sprouting and stabilize the newly formed blood vessels. Endothelial cells directly communicate with pericytes through direct gap junctions and peg-sockets and via paracrine signaling factors to facilitate formation, maturation, and stabilization of the microvasculature.^6^ Pericytes also regulate blood flow by contracting or relaxing to facilitate the high metabolic demand of the retina, especially that of neuronal tissue. Some studies show a role of pericytes in immune cell trafficking by remodeling of the basal lamina or expression of cytokine receptors and toll-like receptors and release of cytokines and chemokines.^90^ The high ratio of pericytes allows for precise control of these functions. Pericyte dysfunction or loss have been reported in various pathologies, for example DR, ischemia, glaucoma, and tumor formation.^4,7,8,20,86,88^ Therefore, restoring pericyte function as a future therapy may well ameliorate the pathological developments.

Further research is required to establish the underlying mechanisms related to pericyte dysfunction and loss and whether manipulating pericytes can be used to treat retinal disorders. With this study, we have visualized pericytes in human retinal flatmounts using IF staining. Using a combination of pericyte markers can shed more light on heterogeneity in the pericyte population. Pericytes are heterogeneous in a tissue- and context-dependent way.^9,73^ Understanding pericyte heterogeneity can provide insights into regional population differences, for example in specific parts of the retina with vascular leakage or angiogenesis. The developed IF staining can be applied to study changes in pericytes in conditions affecting the human retina, such as ischemia in retinal vein occlusions or DR. Staining pericytes in the human retina can provide valuable insight into the pathogenesis of these retinal conditions and can lead to development of new, more effective therapeutic approaches. In the near future, we will apply this newly developed staining protocols to retinas from patients with DR to investigate the changes in pericytes associated with the disease.
